# Pneumococcal Pili Are Composed of Protofilaments Exposing Adhesive Clusters of Rrg A

**DOI:** 10.1371/journal.ppat.1000026

**Published:** 2008-03-21

**Authors:** Markus Hilleringmann, Fabiola Giusti, Barbara C. Baudner, Vega Masignani, Antonello Covacci, Rino Rappuoli, Michèle A. Barocchi, Ilaria Ferlenghi

**Affiliations:** 1 Research Center, Novartis Vaccines and Diagnostics s.r.l., Siena, Italy; 2 Department of Evolutionary Biology, University of Siena, Siena, Italy; Children's Hospital Boston, United States of America

## Abstract

Pili have been identified on the cell surface of *Streptococcus pneumoniae*, a major cause of morbidity and mortality worldwide. In contrast to Gram-negative bacteria, little is known about the structure of native pili in Gram-positive species and their role in pathogenicity. Triple immunoelectron microscopy of the elongated structure showed that purified pili contained RrgB as the major compound, followed by clustered RrgA and individual RrgC molecules on the pilus surface. The arrangement of gold particles displayed a uniform distribution of anti-RrgB antibodies along the whole pilus, forming a backbone structure. Antibodies against RrgA were found along the filament as particulate aggregates of 2–3 units, often co-localised with single RrgC subunits. Structural analysis using cryo electron microscopy and data obtained from freeze drying/metal shadowing technique showed that pili are oligomeric appendages formed by at least two protofilaments arranged in a coiled-coil, compact superstructure of various diameters. Using extracellular matrix proteins in an enzyme-linked immunosorbent assay, ancillary RrgA was identified as the major adhesin of the pilus. Combining the structural and functional data, a model emerges where the pilus RrgB backbone serves as a carrier for surface located adhesive clusters of RrgA that facilitates the interaction with the host.

## Author Summary

*Streptococcus pneumoniae* (pneumococcus) is one of the most important human pathogens and a major cause of morbidity and mortality worldwide, causing respiratory tract infections, community acquired pneumonia, and invasive diseases. Although the pneumococcus is a well-studied bacterial pathogen, first described in the late 19th century, pili on its surface were discovered only recently. Pili are elongated structures extruding from the bacterial surface and were found to be important virulence factors of both Gram-positive and Gram-negative bacteria. Bacterial pili are considered to participate in bacterial adhesion to a host, a crucial step in bacterial infection. In contrast to Gram-negative pili, little is known about the structure of native Gram-positive pili. We used native purified pili of pathogenic pneumococcus TIGR4 to study its structural composition, mainly by the use of cryo EM techniques. Pili were found to be composed of protofilaments that are arranged in a coiled-coil, compact superstructure of various diameters. Adhesive properties of pilus surface located ancillary protein RrgA to selected compounds of the extracellular matrix might be part of the pilus mediated host–pathogen interplay. Analysis of native pneumococcal pili revealed structural basics of a Gram-positive pilus that could also serve as a basis for effective vaccine design.

## Introduction

The Gram-positive bacterium *Streptococcus pneumoniae*, also known as pneumococcus, is one of the most important human pathogens causing respiratory tract infections such as sinusitis, otitis media, and community acquired pneumonia, but also invasive diseases such as septicemia and meningitis. Together with HIV, malaria, and tuberculosis the pneumococcus represents one of the four major infectious disease killers [1]–[4]. Even though pneumococcus is a devastating pathogen, it is also a member of the human commensal flora and is known to asymptomatically colonize the nasopharynx [1]. A major virulence factor of *Streptococcus pneumoniae* is the polysaccharide capsule, by which pneumococci are grouped into at least ninety different serotypes [5]. Other genetic factors, such as CbpA (choline-binding protein A) and pneumolysin, have been described to be of importance for virulence [6]–[8]. Infection by *Streptococcus pneumoniae* leads to invasive disease triggered by initial colonization of the nasopharynx, but the mechanisms of adhesion are not well understood [9]. Recently, pilus harboring pneumococci were discovered and results obtained indicate a key role for these structures in virulence and disease [10],[11]. Furthermore, in a mouse model of intraperitoneal infection Gianfaldoni et al. [12] reported protective immune responses after active and passive immunization with recombinant pilus subunits of *Streptococcus pneumoniae* Type 4 strain TIGR4 (T4). Previously, similar pili-like surface structures had been identified in other Gram-positive bacteria, such as *Corynebacterium diphtheriae* [13],[14], *Actinomyces* spp. [15], group A streptococci (GAS) [16], group B streptococci (GBS) [17] and recently *Mycobacterium tuberculosis* [18] where they were shown to play an important role in the interaction with the host at different stages of infection.

The *Streptococcus pneumoniae* pilus was found to be encoded by the *rlrA* pathogenicity islet [10],[19], initially discovered in T4, a clinical, serotype 4 strain, of which the genome is known [20]. Sequencing of various pneumococcal strains revealed, that not all isolates contain this genetic element [21],[22]. The *rlrA* operon encodes, besides a Rof-A-like transcriptional regulator (RlrA), 3 sortases (SrtB, SrtC and SrtD) and 3 structural proteins RrgA (Swiss-Prot Q97SC3), RrgB (Swiss-Prot Q97SC2) and RrgC (Swiss-Prot Q97SC1) containing a LPxTG motif (or variants thereof) [10],[19],[23].

In contrast to Gram-negative pili, which are composed of non-covalently linked subunits, Gram-positive pili studied so far are thought to be extended polymers formed by a transpeptidase reaction involving covalent cross-linked subunit proteins containing specific amino acid motifs, which are assembled by specific sortases. Sortases are also responsible for the covalent attachment of the pilus to the peptidoglycan cell wall [24]. Fundamental work on this was done by Schneewind and co-workers studying *Corynebacterium* spp. pili [13],[14],[25],[26] and recent reviews summarize the more general knowledge on Gram-positive pili [27]–[29].

In *Corynebacterium diphtheriae*, in addition to a N-terminal signal sequence and a C-terminal cell wall sorting signal, two motifs are considered to be important for the major pilus component, i.e. the so called pilus backbone forming protein: the pilin motif and the so called E-box [13]. Following the corynebacterial system, pneumococcal RrgB was proposed to form the backbone of the pneumococcus T4 pilus structure, as its sequence contains homologues of both motifs. For pneumococcal T4 RrgA and RrgC, a role as ancillary proteins was suggested [10]. These observations are supported by initial electron microscopy (EM) analysis on pneumococcal cells containing pili [10],[11]. Although the precise mechanism of incorporation of RrgA, RrgB and RrgC into the pneumococcal pilus is not yet understood, one hypothesis is that incorporation of the three subunits is specifically catalysed by each of the 3 sortases present in the rlrA islet: in line with this are results found by LeMieux et al. [11] that showed that SrtD is needed for RrgA incorporation into the typical high molecular weight (HMW) structure. In addition, the incorporation of RrgA is dependent on the presence of RrgB but not RrgC.

Whereas genetically based functional studies regarding Gram-positive pili such as those of *Streptococcus pneumoniae* are emerging, structural information of the native entire pilus in Gram-positives is lacking and its significance in infectious disease is not clear. Very recent data based on crystal structures of single pilus subunits of Gram-positive pili in *Streptococcus agalactiae* and *Streptococcus pyogenes* stimulated novel insights into Gram-positive pilus composition [30],[31]. The elucidation of the structure of the native pilus is of great interest not only to increase our understanding of the biology of Gram-positive bacteria, but also as potential tool to develop proper therapeutics and vaccines against pathogenic bacteria like *Streptococcus pneumoniae* [32],[33]. Our approach consists in using native, purified pneumococcal pili of a pathogenic T4 strain to study structure and properties of these Gram-positive surface appendages. We provide for the first time structural evidence of the pneumococcal pilus, which is composed of protofilaments arranged in a coiled-coil superstructure. Structural proteins RrgA, RrgB and RrgC localized to different regions of the same pilus, confirming RrgB as the major compound, followed by clustered RrgA and single RrgC molecules on the pilus surface. RrgA was identified as major adhesion protein towards selected extracellular matrix (ECM) compounds. Structural and functional data indicate the pneumococcal pilus to be a flexible carrier of functional groups able to cross pneumococcal polysaccharide capsule, promoting host cell interaction.

## Results

### Purification and Characterization of Native Pneumococcal T4 Pili

**Figure 1:**
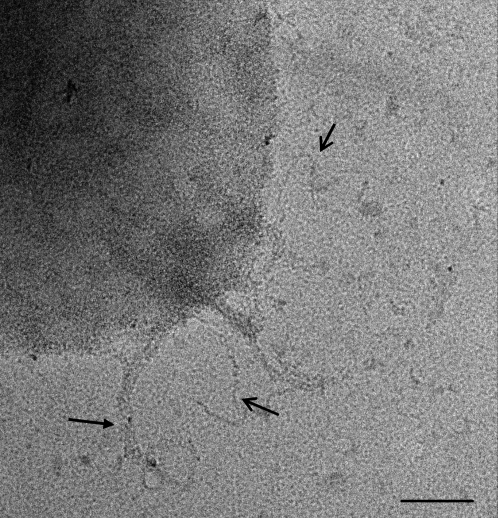
Micrograph of Negative Stained Whole Cell *Streptococcus pneumoniae* TIGR4. Sample stained with 1% buffered phosphotungstic acid (PTA). Open arrows indicate an individual single pilus; arrow indicates bundles of individual pili. Scale bar, 200 nm. (Philips TEM CM200 FEG microscope at 50000× magnification, working at low-dose conditions).

In order to study the pneumococcal pilus in detail, a multi step purification procedure was set up to obtain pure native pili preparations. Pneumococcal pili were isolated from strain T4, bacteria that in low-dose EM showed individual pili and bundles of individual pili on the bacterial cell surface (Figure 1). Both types of appendages were distributed on the bacterial surface, the majority of which (∼65%) belonging to the individual pilus type. The same appearance was found analysing purified pili preparations with cryo electron microscopy (cryo-EM).

**Figure 2:**
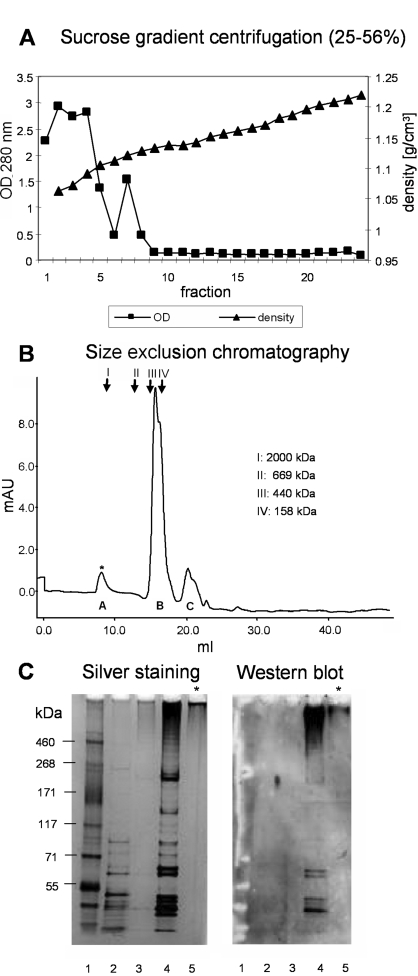
Multi-Step Purification of Native Pneumococcal Pili. SN of mutanolysin treated T4 bacteria was applied to a 25–56% sucrose gradient (A). Pili containing fractions (no. 5–8) were further purified using size exclusion chromatography: HMW pili (peak A*) were separated from lower molecular weight material (peak B and C) (B). Summary of the purification procedure is shown in C (silver stained SDS-PAGE, and respective western analysis with α-RrgB antibodies). Lanes: 1: HMW marker, 2: T4 whole cell lysate, 3: SN mutanolysin digestion, 4: sucrose gradient pool (fractions no. 5–8), 5: HMW pili – size exclusion chromatography pool peak A (*).

Briefly, for pili purification, bacteria were grown on blood agar plates. Harvested bacteria were washed and subjected to mutanolysin treatment. N-acetyl muramidase treatment released pneumococcal pili into the supernatant. Subsequently, supernatants containing pili were applied to a sucrose gradient to separate them from other cellular impurities and to concentrate their relative amount in the sample preparation (Figure 2A). Pili-positive gradient fractions were identified by dot blot analysis with anti-RrgB antibodies. Western analysis (anti-RrgB) of SDS-PAGE separated pili samples was performed to identify HMW material in fraction number 5–8 at the top of the separation gel. Dialysed sucrose pools were concentrated and applied to gel filtration to further purify the pili preparations. As shown in Figure 2B, size exclusion chromatography allowed the separation of HMW pili (peak A) from lower molecular weight material (peak B and C) as proven by SDS-PAGE and western blot analysis with anti-RrgB antibodies (data not shown). As a control, the same purification procedure was performed with a pneumococcus T4 Δ*pil* strain. As expected, HMW pili (peak A) that were eluted in the void volume were not found in the respective delta pilus preparation. A summary of the purification strategy of pneumococcal T4 pili is shown in Figure 2C: Silver stained SDS-PAGE analysis of the different purification steps and the correspondent western blot anti-RrgB analysis show purified HMW pili after gel filtration at the entry of the gel pocket. Purified pili were used for further analysis and to study the structure of pneumococcal pili.

HMW pili, following size exclusion chromatography, were applied to SDS-PAGE and HMW band was subjected to mass spectrometry analysis and N-terminal sequencing (Edman analysis). Tryptic peptide sequence of HMW pili were analysed by MALDI-TOF. Results identifying the cell-wall surface anchor family protein RrgB of *Streptococcus pneumoniae* T4 (gi|15900379) were confirmed by MS/MS analysis: the fragmentation of a peptide with a mass of 2064 Da matched the peptide sequence LAGAEFVIANADNAGQYLAR that is part of pneumococcal T4 RrgB. The Edman analysis resulted in the peptide sequence AGTTTTSVTVHXL, which could be identified as part of T4 protein RrgB. The identified N-terminal starting amino acid corresponds to an alanine, which is located 30 residues downstream of the Met of the RrgB sequence, in agreement with the predicted cleavage site of the signal sequence.

### Composition of Purified Pili: An Analysis of Their Structural Subunits RrgA, RrgB and RrgC by Immunoelectron Microscopy (IEM)

**Figure 3:**
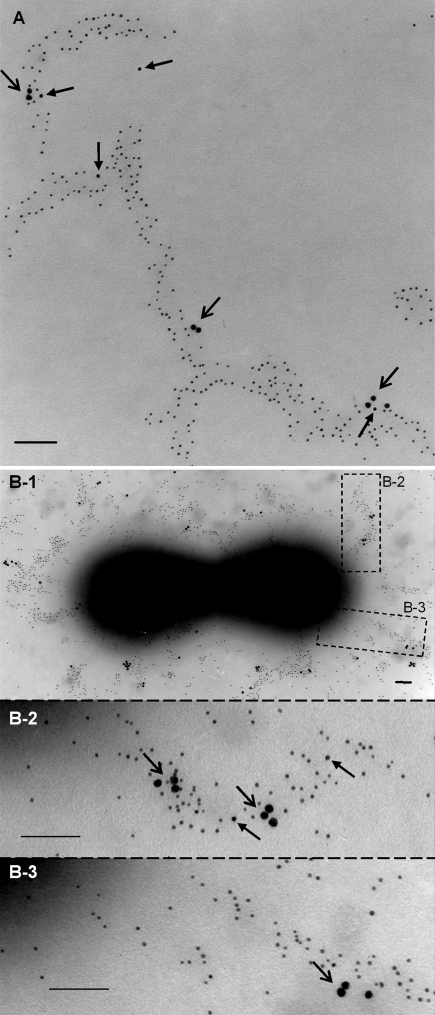
Triple Immunoelectron-Microscopy (IEM) Analysis of the Pilus Subunits of *Streptococcus pneumoniae*. Isolated pili material (A) was incubated with antisera raised against His-tagged RrgA, RrgB and RrgC and conjugated respectively to 15 nm, 5 nm and 10 nm gold particles. The image shows the pilus backbone stained with gold-labelled antibodies raised against the main pneumococcal pilus component (RrgB). Clusters of RrgA ancillary proteins (open arrows) are present along the entire pilus. Single copies of the ancillary protein RrgC (arrows) were found alone or co-localized with the RrgA clusters. Scale bar, 100 nm. The same protocol for triple immunogold EM has been applied to bacteria preparation *of Streptococcus pneumoniae* T4 (B), showing a similar pattern of gold distribution (scale bar, 100 nm).

The detailed composition of pili was investigated by IEM with antibodies raised against recombinant HisTag-RrgA, -RrgB and -RrgC. Initially, single and double IEM were performed with different combinations of the three antisera on both bacteria and isolated pili in order to reveal the presence of all three pilus components. Triple immunogold staining was then performed on the same pilus preparation and on whole bacteria to observe the type of distribution and the relative amount of the 3 structural proteins. Figure 3A shows RrgB distributed evenly along the entire pilus polymer while RrgA and RrgC were present at non-regular intervals along the pilus shaft. An approximate estimation of the relative amounts of the three proteins based on triple, double and single IEM observations indicated that roughly 90% of the gold particles corresponded to RrgB. The remaining 10% circa were composed of RrgA and RrgC, with a higher occurrence of RrgA in comparison to RrgC. In particular, the IEMs showed that RrgA was organized in small clusters, as found by particulate aggregates of 2–3 anti-RrgA antibody units, distributed along the entire pilus surface. Interestingly, RrgC protein was found in single copies and often co-localized with the RrgA clusters. Triple IEM performed on whole bacteria (Figure 3B) confirmed that purified pili conserved the same structural characteristics as native pili attached to bacteria.

### Isolated T4 Pili Are Flexible Structures Representing Morphological Variability

**Figure 4:**
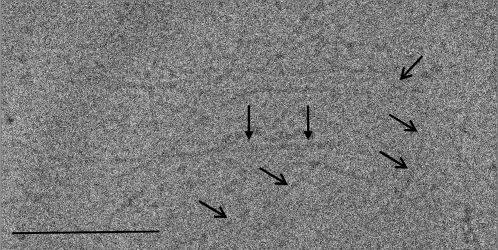
Cryo Electron Microscopy (Cryo-EM) Image of Isolated Single Pili. The image shows the presence of different sized individual pili (open arrow and arrow) distributed on the EM grid where they form a net of elongated structures. Image of the vitrified sample has been taken by cryo-EM low-dose conditions in a TEM CM200FEG microscope at 50000× magnification. Scale bar, 100 nm.

**Figure 5:**
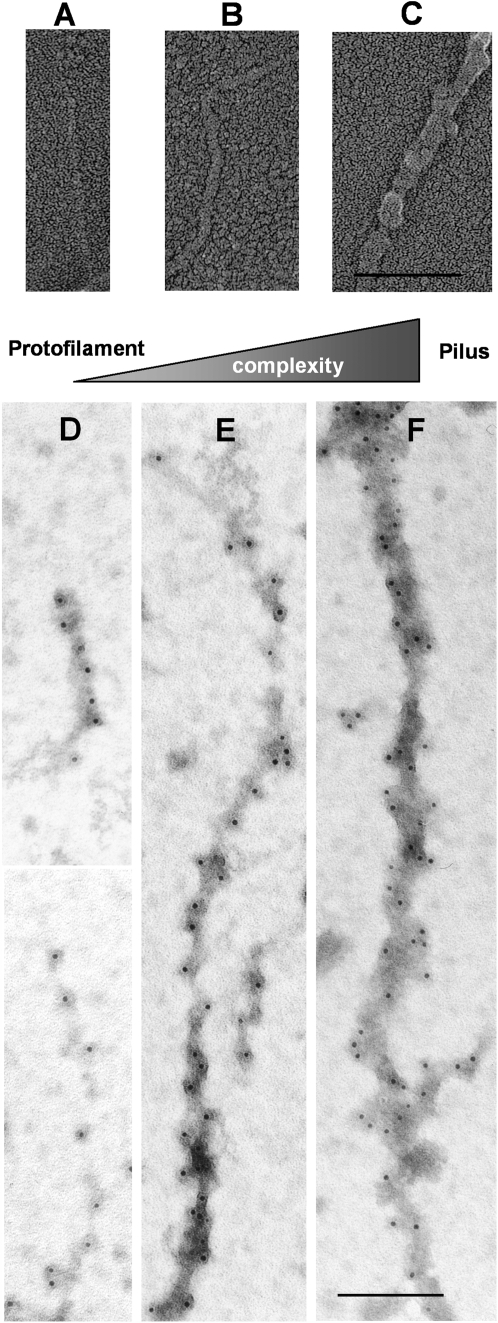
Freeze Drying/Metal Shadowing and Immunogold EM Images of Purified Pili Preparation. Gallery of shadowed (A, B and C) and immunogold labelled (D, E and F) pili showing the diversity of pili morphology and sizes. The protofilament is visible in (A and D), pili are visible in (B, C, E and F). For IEM isolated pili material was incubated with antisera raised against His-tagged RrgB followed by secondary gold antibodies. The metal shadow underlines the increased complexity when going from protofilaments to pili. Scale bar, 100 nm.

**Figure 6:**
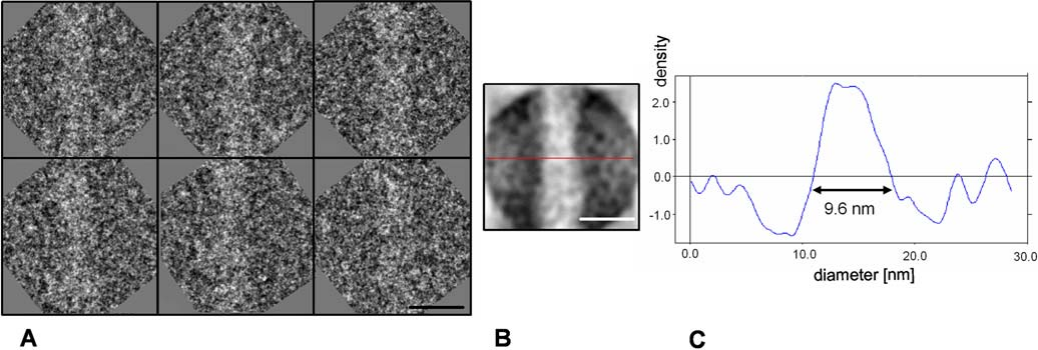
Density Profile of Thin Individual Pili. (A) Six short boxed thin pili extracted from micrographs containing purified single pili. Scale bar, 10 nm. (B) Averaged thin pili after translational and rotational alignment of the boxed regions. Scale bar, 10 nm. (C) Density profiles across the pili axis of the averaged thin pili projected onto the short axis. Calculated average diameter (double-headed arrow) for thin pili is 9.6±0.3 nm.

Purified HMW pili preparations, observed by cryo-EM (Figure 4) and freeze drying/metal shadowing techniques (Figure 5A, 5B and 5C) showed that pili were elongated structures of up to 1 µm in length. They were identified as elongated and adhesive structures with the tendency to form a net on the EM grids (Figure 4). Generally present as individual single pili of different thickness, they were also found to form bundles of individual pili (data not shown). Among the individual single pili several classes could be identified based on their diameter and morphological variability (Figure 5). The most subtle filaments (Figure 5A and 5D) showed a linear morphology with no evident periodicity. A corresponding IEM displayed a linear distribution of gold particles binding to RrgB backbone proteins (Figure 5D). We defined this type of filament as the pilus protofilament. Increasing filament widths (Figure 5B and 5C) resulted in an increasing complexity of the filaments, as clearly indicated by the higher number of gold particles decorating the filaments in a non-linear spacing (Figure 5E and 5F). The majority of the pili could be assigned to the class of thin pili (∼63%) (Figure 5B and 5E) with an observed average diameter of 9.5 nm, as calculated from cryo-EM data (Figure 4). The remaining ∼37% of the isolated pili were of larger diameters, the majority of which, individualised as class of thick pili, showed a width of about 10.5 nm (data not shown). Further structural analyses were performed on the thin pili, more than 200 individual thin pilus segments were selected [34] from digitized micrographs (Figure 4) obtained by cryo-EM on vitrified samples by using 300×300 pixel size boxes. Thus working with shorter segments that resulted to be approximately straight for the chosen box size was possible. Pili segments were treated as discrete single particles, 300 pixel in length and processed [35] by first aligning them rotationally and translationally to a reference cylinder centered into the image box. All segments that did not align with the reference were eliminated. Finally 124 segments of the thin pili (Figure 6A) were kept and used to generate averaged thin pili segments with an increased signal-to-noise ratio (Figure 6B). Subsequently the diameter of the averaged thin pilus segments could be calculated from its density profile, by creating the profile (IMAGIC5) [35] of the averaged segment generated from the 2D image of the pilus (red line). The results showed a diameter of 9.6±0.3 nm for the thin pilus. Moreover the shape and the values of the density profile clearly indicated that thin pili were rather compact structures.

### T4 Pili Are Coiled-Coil Superstructures Composed of Protofilaments

**Figure 7:**
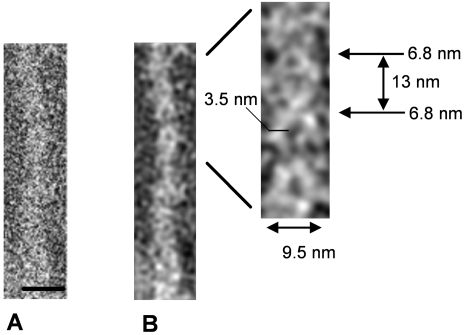
Images of Gaussian Filtered Thin Individual Pili Show Their Structural Composition. (A) Images of original pilus taken at low dose conditions. (B) Gaussian filtered thin individual pili show their structural composition. Inset reveals an enlarged view of the thin pilus corresponding to the pilus crossover. Crossover position (arrow) for thin pilus is indicated. The protofilament diameter was measured as 3.5 nm. Crossover diameters (arrows) were calculated as 6.8 nm for thin pili (9.5 nm diameter). Scale bars, 10 nm.

Interestingly when a double Gaussian filter, where both, high and low frequency transmission were cut off, was applied on original thin pili data (Figure 7A), the filtered 2D image (Figure 7B), clearly showed that thin individual pili were composed of at least two protofilaments arranged in a coiled-coil superstructure. The Gaussian filtered image of the thin pilus showed zones where the 3.5 nm diameter protofilaments were tightly intersected (crossovers), resulting in a pilus diameter of 6.8 nm. This was alternated by zones where protofilaments had a more relaxed intersection with a pilus diameter of 9.5 nm. The average distance between two neighbouring crossovers was approximately 13 nm. Preliminary results suggest that thick pili are also composed of similar protofilaments arranged in coiled-coil manner (data not shown).

### RrgA and Purified Pili Bind to Selected Extracellular Matrix Components *In Vitro*

**Figure 8:**
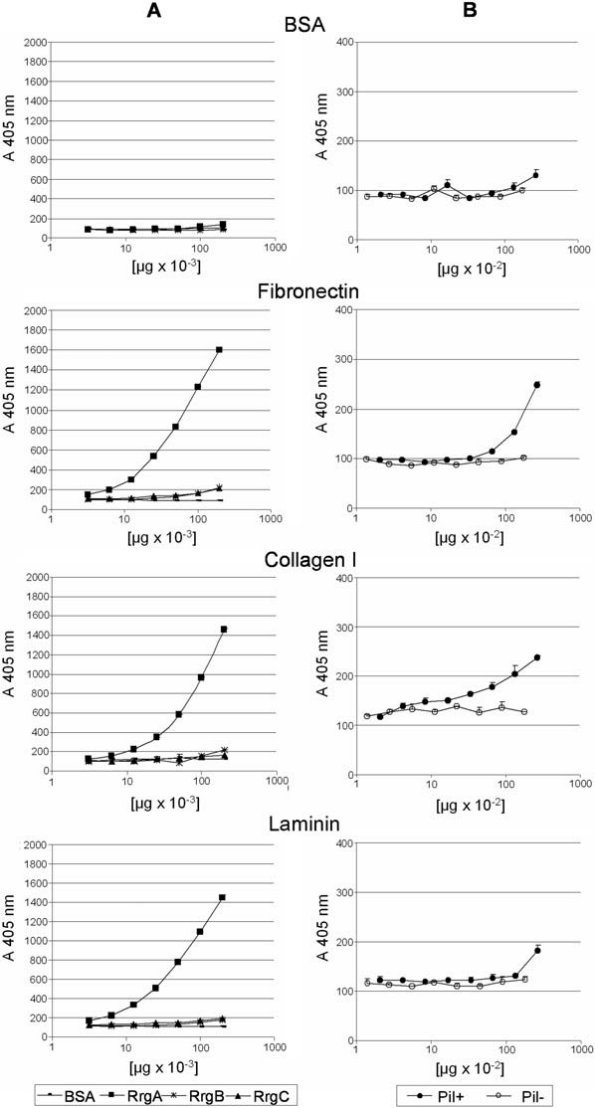
Dose Dependent Binding of RrgA to Selected Extracellular Matrix (ECM) Components. Shown are the results of binding increasing concentrations of purified T4 pilus proteins HisTag-RrgA, -RrgB and -RrgC (A) and HMW pilus preparations (B) to fibronectin, collagen I and laminin. BSA and delta pilus mock preparation served as negative controls. Binding was quantified by ELISA at an absorbance of 405 nm. Points represent the means (error bars, standard errors of the means) of measurements made in triplicate.

In order to investigate adhesive properties of isolated pili and of single pilus subunits RrgA, RrgB and RrgC *in vitro* binding assays were performed to study the interaction to selected ECM components using this approach to provide a proof in principle [36],[37]. In particular, fibrinogen, fibronectin, laminin, lactoferrin and collagen I were selected, as these cellular compounds are known to be recruited by pathogenic agents [9], [38]–[40]. Apart from overall pili adhesive properties special interest was drawn to a potential role of distinctly surface exposed RrgA and RrgC. For this purpose, serial diluted samples of recombinant proteins RrgA, RrgB and RrgC, as well as native purified pili and a pilus negative control were added to 96-well plates coated with the selected ECM components. Binding was detected using polyclonal sera raised against the single recombinant pilus subunits and quantified by enzyme-linked immunosorbent assay (ELISA). As demonstrated in Figure 8 (lane A), RrgA showed very pronounced dose depending binding to most of the tested ECM compounds, whereas binding results obtained for RrgC and RrgB are negligible. In addition, RrgA binding was observed for lactoferrin and fibrinogen whereas no binding was detected to vitronectin coated plates (data not shown). Bovine serum albumin (BSA) was used as negative control in all the assays. Binding studies performed using purified pili (Figure 8, lane B) showed binding to ECM components clearly distinguishable from the *Streptococcus pneumoniae* delta pilus negative control.

## Discussion

Pili are considered important key players in bacterial pathogenesis and disease [10],[27]. To date structural information of the native pilus in Gram-positive bacteria is lacking, therefore the elucidation of their structure and function are of great interest. Our approach consisted in obtaining native purified pili from a pathogenic strain of *Streptococcus pneumoniae* to study pilus structure and function. Special emphasis was drawn on the overall structural principle of the native pilus and the role of the individual structural proteins RrgA, RrgB and RrgC. As opposed to pili attached to the bacterial surface, isolated pili allow a broader spectrum of analyses and at the same time permit a comprehensive characterization of their structure in sufficient detail to describe the function at the quasi-molecular level. We developed a multi step purification procedure to obtain native pilus material that allowed to perform the desired analyses.

T4 bacteria were examined by low dose negative stain EM, IEM and cryo-EM, showing that the bacterial surface is covered with elongated, flexible and rather sticky pilus-like appendages of up to 1 µm long. Interestingly, we observed pili of various morphologies: individual single pili, distinguishable into different classes by their diameter (ranging from 9.5 nm up to 10.5 nm), and bundles of individual pili. Whether or not this has a physiological role has yet to be evaluated. The established purification method allowed for the isolation of pure HMW material that showed pili morphotypes having the same features as those found for wild-type pili expressed on whole bacteria. Structural analysis based on cryo-EM data of vitrified, purified single pili revealed that they are organised in coiled-coil superstructures made by at least two protofilaments. The observed range in pilus diameters could either reflect a difference in the degree of packaging of the identical protofilaments into the pili superstructures or a higher number of protofilaments composing the larger pili. The protofilaments of the thin pilus type are organized to form a rather compact superstructure. However no distinct internal cavity could be identified within the thin pilus structures. Preliminary results on the thick type of pilus suggest also a protofilament based structure.

The picture of the individual pili that emerges from our analysis indicates that the *Streptococcus pneumoniae* pilus does not exist in a single structural state but rather in several structural states that are underlaying, among other things, the flexibility and elasticity of these polymers while keeping the same protein composition and proteins roles: RrgB forming the backbone, surface located clustered RrgA being the major ancillary protein involved in adhesion and RrgC as minor ancillary protein of still unknown role.

Additional biochemical analysis of isolated pili supports RrgB as the main pilus building block: Mass analysis of native pili revealed clear signals only for peptides that could be assigned to structural protein RrgB. Neither RrgA nor RrgC related signals could be identified, which is probably due to their minor abundance and the overall hindered protease digestibility of the isolated HMW pili. Similarly, the determination of the N-terminal amino acid sequence of the purified pili by Edman analysis, matched only with the sequence following the predicted signal sequence of RrgB. The observation that purified pili show a free N-terminal part of RrgB starting exactly after the signal sequence may reflect properties of pilus biosynthesis and subsequently its structure. Purified pili of a *Streptococcus pneumoniae* delta RrgA background show a similar overall pili structure composed of protofilaments. This is in accordance with studies showing that a pneumococcal RrgA mutant strain is still able to form pili, whereas a Δ*rrgB* Δ*rrgC* strain is not [11] and fits with the detected structural organisation of RrgA clusters on a coiled-coil RrgB based scaffold.

Gram-positive and Gram-negative pili differ substantially in their assembly mechanisms (Gram-negative pili: non-covalently linked protein subunits versus Gram-positive pili: covalently linked subunits), interestingly both types of bacterial pili share a common arrangement, the coiled-coil superstructure. Our work supports that, also for Gram-positive bacteria, adhesive pili extending from the bacterial surface are the most appropriate structures to promote biological function like adherence to the host due to their structural arrangement leading to flexibility and elasticity. Until now this could be only observed in Gram-negative bacteria like *Haemophilus influenzae* type b pili and *Escherichia coli* P-pili [41] or *Actinobacillus actinomycetemcomitans* [42]. Results by Kang et al. [30] identified a novel principle of stabilization of long and thin pilus filaments by isopeptide linkage between pilus subunits of Gram-positive *Streptococcus pyogenes*. Further work will have to show whether similar design can also be found in other Gram-positive pili, like those of *Streptococcus pneumoniae*. Our results suggest that the coiled-coil arrangement of the protofilaments, forming the pneumococcal pili, might be an additional principle, other than isopeptide bond formation, to confer stability and flexibility to subtle surface structures in order to withstand mechanical rigors outside the cell.

Research on bacteria and therefore also the study of the pneumococcal pilus should be seen in the context of bacterial life cycles within specific ecological niches and e.g. in the interaction with its host. Pneumococcal infection of the host occurs mainly via the mucosal route [1], thus bacteria need to develop strategies to adhere and resist actions of the human immune system like mucosal clearance [43]. Studies performed using pilus negative mutants of T4 clearly demonstrate a positive correlation between bacterial virulence and colonization and the presence of the pilus [10]. We therefore wanted to study, if the structural data found for the isolated pili help us to better understand the functionality of pilus mediated pneumococcal behaviour within a host, and whether structural properties of the pneumococcal pilus could be function derived.

**Figure 9:**
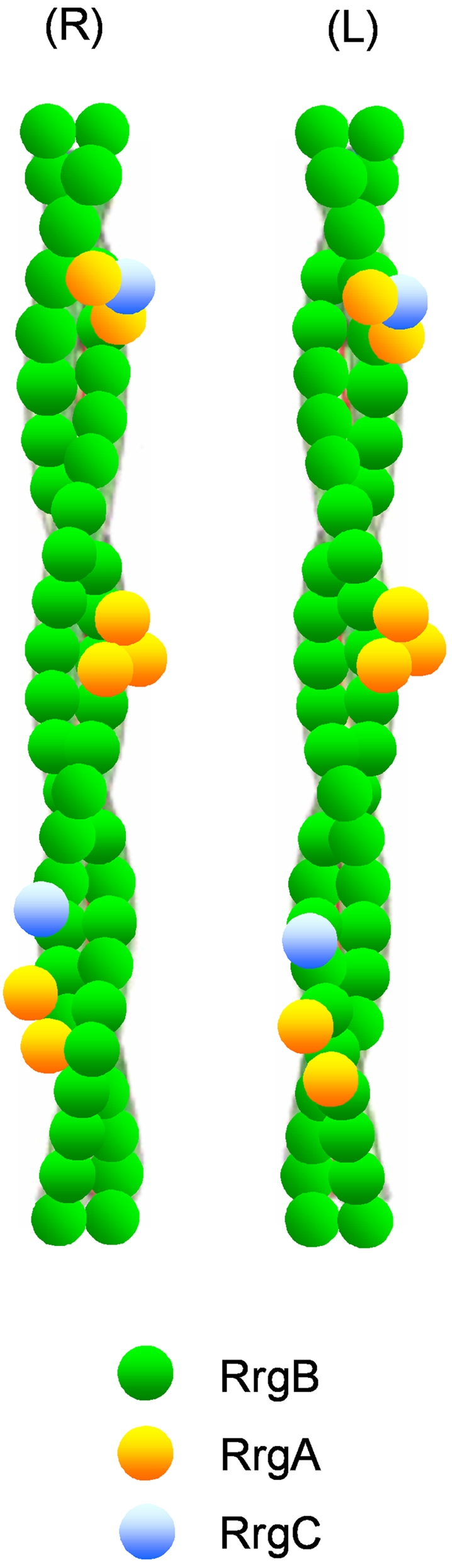
Model of a Pneumococcal Pilus. Model showing T4 pneumococcal pilus composed of at least two RrgB protofilaments (green) arranged in a coiled-coil superstructure with surface located ancillary proteins (RrgA and RrgC) is based on cryo-EM, freeze drying/metal shadowing EM, IEM and biochemical data. (R) and (L) illustrate a possible right and left handed orientation of the thin pilus respectively. Outlines are not drawn to scale.

How does the pneumococcal pilus mediate interaction with its host? Our data suggest that pneumococcal pili are flexible protofilament-based structures composed of ancillary proteins RrgA and RrgC and the RrgB backbone (Figure 9). Recently, proteins of group B streptococcal pilus were found to facilitate the interaction with endothelial cells [44]. Our data elucidate the adhesive properties of RrgA to fibronectin, laminin and collagen, suggesting that the clusters containing RrgA are the adhesive regions of pili. In silico analysis of RrgA (T4) sequence identified domains important for adhesion, like MSCRAMM motifs [19] and homologues of the von Willebrand factor A (vWFA) [45]. Interestingly, PapG, the adhesin of *Escherichia coli* P-pili, that binds to uroepithelial cells in its human host was also found to be located on the pilus surface, but only at the very distal end of the pilus fiber [46]. *Streptococcus pneumoniae* is a mucosal commensal, a mucosal pathogen and an invasive pathogen. Colonization of the nasopharynx by *Streptococcus pneumoniae* is a prerequisite for the development of pneumococcal disease and the result of a complex interplay between host and pathogen factors. Respiratory pathogens are known to release products which interfere with mucosal defences, causing epithelial disruption and cell death [47],[48]. *Streptococcus pneumoniae* was seen to adhere in particular to damaged cells and extruded cells [47], and bacteria were often found to be associated with damaged epithelium and exposed ECM [49]. Pathogen-ECM interactions have been found to be associated with adhesion and subsequent invasion of the pathogen [9]. Adhesive properties of pilus surface located ancillary protein RrgA to selected compounds of the ECM might therefore be part of the pilus mediated host-pathogen interplay. Flexibility of the pilus, as suggested by the protofilament-based structure, supports its functionality under *in vivo* conditions. Interestingly, recent work done by Nelson et al. [50] identified adhesive properties of pneumococcal pilus RrgA in cell-based assays. This together with data showing the impact of RrgA on pneumococcal virulence in mice [19],[50] indicate that the polypeptide may function at more than one stage in the infection process.

In summary, this report presents support for the structural composition of the *Streptococcus pneumoniae* pilus as an oligomeric appendage with adhesive properties and future work will help to further improve our understanding of the structure and function of the pilus and its main components.

## Materials and Methods

### Bacterial strains, cell lines and culture conditions

*Streptococcus pneumoniae* type 4 strain TIGR4 has been described [20]. Mutants TIGR4Δ*pil* (*rrgA-srtD*) [10] and TIGR4Δ(*rrgA*) [50] were kindly donated by B. Henriques-Normark (Karolinska Institute, Stockholm). The pneumococcal strains were stored at −80°C in 12% glycerol and routinely grown at 37°C in 5% CO_2_ on Tryptic Soy Agar (Becton Dickinson) supplemented with 5% defibrinated sheep blood or in Tryptic Soy Broth (Becton Dickinson). When appropriate, erythromycin (Sigma-Aldrich) as selection marker was used.

### Expression and purification of RrgA, RrgB and RrgC

Standard recombinant DNA techniques were used to construct all expression plasmids. Vector pET 21b+ was purchased from Invitrogen. Full length sequence of T4 pili proteins RrgA (TIGR annotation No. sp0462), RrgB (TIGR annotation No. sp0463) and RrgC (TIGR annotation No. sp0464) with exception of their N-terminal signal sequence and C-terminal cell wall sorting signal motif, hydrophobic stretch and charged tail was cloned into pET21b+: pellets of IPTG induced recombinant *Escherichia coli* BLR(DE3) cultures, containing expressed His-tagged RrgA, RrgB and RrgC proteins respectively, were subjected to lysis by lysozyme in a BugBuster (Novagen), Benzonase Nuclease (Novagen) solution containing proteinase inhibitors. After centrifugation at 35000 rpm for 1 h at 4°C, the soluble fraction was subjected to metal chelate affinity chromatography on His-Trap HP columns (GE Healthcare) equilibrated and eluted according to manufacturer's instructions. Pooled fractions were dialysed overnight (ON) against 0.9% NaCl and stored at −80°C until further use. Protein concentration and purity was determined by scanning densitometry of Coomassie Blue-stained SDS-PAGE using a BSA standard and measuring 280 nm absorption of the protein solution (NanoDrop®).

### Native T4 pili purification

*Streptococcus pneumoniae* T4 was chosen as starting material as far as the bacteria belong to a clinical relevant serotype 4 isolate, the sequence of which is known [20] and it represents a well characterized pneumococcal strain.

*Streptococcus pneumoniae* T4 glycerol stock (−80°C) was grown on Tryptic Soy Agar supplemented with 5% defibrinated mutton blood (ON at 37°C in 5% CO_2_). Fresh bacteria were used to incubate new agar plates and cultivated for about 12 h (at 37°C in 5% CO_2_). Harvested bacteria of about 10 plates were washed once in 35 ml PBS, and resuspended in 2 ml protoplast buffer PPB (10 mM MgCl_2_, 50 mM NaPP_i_ pH 6.3, 20% sucrose) containing protease inhibitors. About 450 U of mutanolysin in 100 mM NaPP_i_ pH 6.3 were added to each half of the suspension and incubated at 37°C for about 5 to 8 h with gentle shaking until protoplast formation was detected (microscopic control). Supernatant, containing digested pilus material was loaded on a sucrose gradient (25 to 56% in 10 mM MgCl_2_, 50 mM NaPP_i_ pH 6.3) and run for about 20 h at 38000 rpm (4°C). All further steps were performed at 4°C using buffers containing protease inhibitors. In addition, benzonase nuclease (Novagen) was added to remove DNA and RNA impurities. Collected gradient fractions were tested for pilus material using anti-RrgB antibodies. Pilus containing fractions were pooled and dialyzed against 10 mM MgCl_2_, 50 mM NaPP_i_ pH 6.3 for about one day to remove sucrose. When necessary, additional chromatography steps were added to reduce polydispersity and pooled pilus preparations were concentrated before loading them on a Superose 6 10/300 GL column (Amersham Biosciences). Gel filtration resulted in separation of pilus containing material of different molecular weight. Purified pilus fractions were judged to be homogeneous based on EM and SDS-PAGE. Samples were stored at −80°C or liquid nitrogen until further use.

### In-gel protein digestion and sample preparation for MS analysis

Protein spots corresponding to HMW pili material were excised from SDS-PAGE gels (3–8% TA, Invitrogen), washed with 100 mM ammonium bicarbonate/ACN 50/50 v/v, and dried using a SpeedVac centrifuge (Savant, Holbrook, NY, USA). Dried spots were digested for 2 h at 37°C in 12 ml of 0.012 µg/ml sequencing-grade modified trypsin (Promega, Madison, WI, USA), in 50 mM ammonium bicarbonate. After digestion, 5 µl of 0.1% Trifluoroacetic acid (TFA) were added, and the peptides were desalted and concentrated with Zip-Tips (C18, Millipore). Samples were eluted with 2 µl of 5 g/l 2,5-dihydroxybenzoic acid in 50% ACN/0.1% TFA onto the mass spectrometer Anchorchip 384 (400 µm, Bruker Daltonics, Bremen, Germany), and allowed to air-dry at room temperature. MALDI-TOF spectra were acquired on a Bruker Ultraflex MALDI-TOF instrument (Bruker Daltonics). Protein identification was carried out by both automatic and manual comparison of experimentally generated monoisotopic values of peptides in the mass range of 700–3000 Da with computer-generated fingerprints using MASCOT software running on proprietary databases. Identifications were confirmed by MS/MS analysis: after denaturing the samples in a MS-compatible detergent (RapiGest SF, Waters) and boiling for 15 min, in-solution digestion was performed by adding 2 µg of trypsin, and allowing digestion ON. MS/MS spectra were acquired using an ESI-q-TOF Micro mass spectrometer (Waters), coupled to a nano-LC on a CapLC HPLC system (Waters). A MS survey scan was used to automatically select multicharged peptides over the m/z range of 400–2000 for further MS/MS fragmentation. After data acquisition, the MS/MS spectra were combined, smoothed and centroided by MassLynx software, version 4.0 (Waters). Search and identification of peptides were performed with a licensed version of MASCOT, in a local database, after converting the acquired MS/MS spectra in .pkl files.

### N-terminal sequencing (Edman degradation) of HMW purified pili

Identification of the N-terminal amino acid sequence of HMW pili material by Edman degradation was performed according to standard conditions. HMW pili material, following size exclusion chromatography, was applied to SDS-PAGE (3–8% TA; Invitrogen). After western transfer to PVDF membrane, HMW pili band was cut out and used for Edman analysis.

### SDS-PAGE and western analysis

SDS-PAGE analysis was performed using NuPAGE™ 3–8% Tris-Acetate Gels (Invitrogen) according to the instructions of the manufacturer. HiMark™ pre-stained HMW protein standard (Invitrogen) served as protein standard. Western analysis was done using standard protocols. Antibodies against recombinant HisTag-RrgB were used at 1/10000 dilution. Secondary goat anti-mouse HRP antibodies were used at 1/30000.

### Animal sera and antibodies

Antibodies against recombinant HisTag-RrgA (mouse; guinea pig), -RrgB (mouse), and -RrgC (mouse; rabbit) were produced in our lab and tested for specificity. Secondary goat anti-mouse HRP antibodies were obtained from Bio-Rad. Gold labelled antibodies for IEM were purchased of BBInternational: anti-mouse (5 nm), anti-rabbit (10 nm) and anti-guinea pig (15 nm).

### ELISA

96-well MaxiSorp™ flat-bottom plates (Nunc, Roskilde, Denmark) were coated for 1 h at 37°C followed by an ON incubation at 4°C with 2 µg/well of respective ECM vitronectin (from human plasma, BD Biosciences), lactoferrin (from human milk, Sigma), collagen I (from human lung, Sigma) and fibrinogen (from human plasma, Sigma) and with 1 µg/well with laminin (from human placenta, Sigma) and fibronectin (from human plasma, Sigma) in phosphate-buffered saline pH 7.4 (PBS). A BSA coated plate served as negative control. Plates were washed 3 times with PBS/0,05% Tween 20 and blocked for 2 h at 37°C with 200 µl of PBS/1% BSA followed by 3 washing steps with PBS/0,05% Tween 20. Recombinant protein samples (HisTag-RrgA, -RrgB and -RrgC) were initially diluted to 4 µg/ml with PBS. 200 µl of protein solution or 100 µl of wild type pilus preparation (53 µg/ml) and 100 µl T4Δ*pil* sample (35 µg/ml), diluted in 200 µl total volume with PBS, and respective controls were transferred into coated-blocked plates in which the samples were serially diluted two-fold with PBS, obtaining a final volume of 100 µl/well. Plates were incubated for 2 h at 37°C and ON at 4°C. The plates were washed 3 times and incubated for 2 h at 37°C with respective primary mouse anti-HisTag-Rrg antibodies (1/10000 dilutions); pilus coated plates were incubated with anti-HisTag-RrgB antibodies. After another 3 washing steps, antigen-specific IgG was revealed with alkaline phosphatase-conjugated goat anti-mouse IgG (Sigma Chemical Co., SA Louis, Mo.) after 2 h of incubation at 37°C, followed by addition of the phosphatase alkaline substrate p-nitrophenyl-phosphate (Sigma). Read out was performed at 405 nm by an ELISA plate reader.

### Immunogold labelling and EM

Formvar-carbon-coated nickel grids were charged with 5 µl of purified sample and let stand for 5 min. The grids were then fixed in 2% paraformaldehyde (PFA) in Phosphate Buffered Saline 0.1 M pH 7.4 (PBS) for 5 min, and placed in blocking solution (PBS containing 1% normal rabbit serum and 1% BSA) for 1 h at room temperature. The grids were then floated on drops of polyclonal antibodies α-RrgA (guinea pig), α-RrgB (mouse) and α-RrgC (rabbit) at dilution of 1∶10 in blocking solution for 1 h at room temperature, washed with 5 drops of blocking solution for 5 min, and floated on secondary gold-conjugated antibodies (goat anti-mouse IgG, 5 nm; goat anti-rabbit IgG, 10 nm; goat anti-guinea pig IgG, 15 nm) diluted 1∶20 in blocking buffer for 1 h. The grids were then washed with five drops of PBS and fixed in 2% PFA/PBS for 5 min at room temperature. Finally samples were washed with 8 drops of distilled water. Grids were stained with 1% buffered phosphotungstic acid (PTA) (pH 6.5) for 15 s, the excess of solution was soaked off by Whatman filter paper. The grids were examined in a CM10 Transmission Electron Microscope (TEM, Philips Electronic Instruments, Inc) operating at 80 kV. The command boxer from software EMAN was used to isolate and to count the single gold particles of different sizes.

### Freeze drying/metal shadowing

20 µl of the solution containing purified pili were transferred onto a cover slip that had been previously cleaned by immersion in chromic acid solution followed by several rinses in distilled water. Pili were allowed to sediment on the glass surface then the cover slips were rinsed in distilled water to remove the excess of material. Immediately before freezing each cover slip was rapidly rinsed in distilled water, and a thin meniscus of solution was left on the glass to prevent dehydration of the samples. While the freezing machine was brought to its lowest temperature 4^o^K, the tiny glass was placed onto a thin slice of aldehyde fixed lung for support during freezing. This was accomplished by slamming the samples onto the liquid helium-cooled copper block of a quick freezing device (Cryopress; Med-Vac, Inc., St. Louis, MO). The frozen samples were freeze dried in a freeze etching unit (Baf 301; Balzers S.p.A., Milan, Italy) for 20 min at −80°C. Pilus absorbed to the cover slip were rotary replicated with ∼2 nm of platinum applied from an angle of 24° above the horizontal and then backed with 25-nm-thick film of pure carbon. Replicas were separated from the glass by immersion in concentrated hydrofluoric acid then cleaned with sodium hypochlorite. After several rinses in distilled water replicas were picked up on 75-mesh formvar-coated microscope grids. Samples were viewed in a transmission electron microscope (CM10; Philips Electronic Instruments, Inc, Mahwah, NJ) operating at 80 kV.

### Low dose EM – negative staining

5 µl aliquots of whole bacteria were applied to 200-mesh copper grids coated with a thin carbon film and let stand for 5 min. The grids were first washed by streaming several drops of PBS over the grids. They were subsequently negatively stained by two drops of 1% buffered PTA (pH 6.5). The last drop was left on the grids for 17 s. Finally the grids were washed with several drops of ddH_2_O, the excess of liquid was soaked off by Whatman filter paper and quickly air dried. The grids were observed using a CM200 FEG Philips Electron Microscope (FEI, Eindhoven, The Netherlands), equipped with a GATAN GIF 2002 postcolumn energy filter (Gatan, Pleasanton, California, United States), and images were collected at an accelerating voltage of 200 kV and a nominal magnification of 50000×, on Kodak SO163 film.

### Cryo-EM

5 µl of purified pili preparation were loaded onto a glow discharged Quantifoil holey carbon grid with 2 µm holes. After being blotted from the front side with a slip filter paper (Whatman No. 4), the grid was flash frozen into liquid ethane as described [51].

### Image processing

Micrographs taken at 50000× of magnification were digitized on a IMACON 949 scanner at spacing of 7.95 µm resulting in a nominal sampling of 1.6 Å/pixel-1. Pili were picked from digitized images using the command “helixboxer” from the software EMAN [34]. Digitized pili images were cut into individual repeats by using boxes of 300×300 pixels, with overlapping ends, using 10 pixel shift for each box, so that adjacent boxes had 90% overlap. The isolated repeats were treated as single particles. In a first analysis, the straightest pili segments were selected and pre-aligned interactively, subsequently the pre-aligned repeats were aligned using alignments with only limited angular ranges (−5°, +5°), finally a vertical alignment has been performed using as a future-less reference the projection of a model cylinder followed by translational alignment perpendicular to the cylinder axis only. Aligned repeats were than subjected to high-pass and low-pass filtrations before the density profiles were calculated (the densities across the filament axis of the pili were projected onto the short axis) using different commands of IMAGIC 5 [35] and of Bsoft software [52]. All the aligned and filtered images were consistent: they all presented centred rods with similar diameters. The only major differences were the surrounding stain distributions.

### Accession numbers

Swiss-Prot (http://www.expasy.org/sprot/) accession numbers for pilus proteins mentioned in the text are:

SP_0462, RrgA (TIGR4) Q97SC3

SP_0463, RrgB (TIGR4) Q97SC2

SP_0464, RrgC (TIGR4) Q97SC1
